# Human Milk Oligosaccharides: A Comprehensive Review towards Metabolomics

**DOI:** 10.3390/children8090804

**Published:** 2021-09-14

**Authors:** Laura Corona, Anna Lussu, Alice Bosco, Roberta Pintus, Flaminia Cesare Marincola, Vassilios Fanos, Angelica Dessì

**Affiliations:** 1 Department of Surgical Sciences, University of Cagliari and Neonatal Intensive Care Unit, AOU Cagliari, 09124 Cagliari, Italy; laucoron@gmail.com (L.C.); annalussu94@gmail.com (A.L.); gomberta@icloud.com (R.P.); vafanos@tiscali.it (V.F.); angelicadessi@unica.it (A.D.); 2 Department of Chemical and Geological Sciences, University of Cagliari, Cittadella Universitaria, SS 554, km 4.5, Monserrato, 09042 Cagliari, Italy; flaminia@unica.it

**Keywords:** metabolomics, human milk oligosaccharides, human milk, secretor phenotype, breast milk

## Abstract

Human milk oligosaccharides (HMOs) are the third most represented component in breast milk. They serve not only as prebiotics but they exert a protective role against some significant neonatal pathologies such as necrotizing enterocolitis. Furthermore, they can program the immune system and consequently reduce allergies and autoimmune diseases’ incidence. HMOs also play a crucial role in brain development and in the gut barrier’s maturation. Moreover, the maternal genetic factors influencing different HMO patterns and their modulation by the interaction and the competition between active enzymes have been widely investigated in the literature, but there are few studies concerning the role of other factors such as maternal health, nutrition, and environmental influence. In this context, metabolomics, one of the newest “omics” sciences that provides a snapshot of the metabolites present in bio-fluids, such as breast milk, could be useful to investigate the HMO content in human milk. The authors performed a review, from 2012 to the beginning of 2021, concerning the application of metabolomics to investigate the HMOs, by using Pubmed, Researchgate and Scopus as source databases. Through this technology, it is possible to know in real-time whether a mother produces a specific oligosaccharide, keeping into consideration that there are other modifiable and unmodifiable factors that influence HMO production from a qualitative and a quantitative point of view. Although further studies are needed to provide clinical substantiation, in the future, thanks to metabolomics, this could be possible by using a dipstick and adding the eventual missing oligosaccharide to the breast milk or formula in order to give the best and the most personalized nutritional regimen for each newborn, adjusting to different necessities.

## 1. Introduction

Nutritional, immunological, and developmental benefits provided by human milk have been widely described in the literature as exquisite tools towards a “personalized medicine” [1]. Indeed, the composition of human milk adapts to the nutritional and non-nutritional needs of infants during lactation [2], thus resulting in a unique, tailored, and dynamic bio-fluid which is considered to be the golden standard of infants’ nutrition in general if the mother and the infant are healthy [3,4,5,6]. 

Regarding its composition, the first research on human milk oligosaccharides (HMOs) dates back to 1954, when Polonowski and Montreuil applied two-dimensional paper chromatography to obtain oligosaccharides from the so-called carbohydrate “*gynolactose*” [7]. Since then, there has been a growing interest in human milk glycobiome, as witnessed by the consistent amount of evidence concerning the biological properties, the physiological function and epigenetic roles of HMOs. As said by Lars Bode, one of the main experts in this field, HMOs “*are more than just food for bugs*”. Indeed, not only do they serve as prebiotics, stimulating the development of a favorable microbiota, they also act as antiadhesive and antimicrobial factors [8]. Moreover, they correlate with a lower risk of gastrointestinal, respiratory and urinary tract infections [9]. Strong benefits come from the bioactive role of these metabolites in neurocognitive development as well [10]. HMOs are also believed to program the infant immune system, thus protecting against the risk of developing allergies and autoimmune diseases [11,12].

A major challenge of our time, as pediatricians, is to guarantee these inestimable advantages to newborns and infants who cannot benefit from breastfeeding but also to those breastfed infants whose mother’s milk might lack some HMOs, such as 2′-FL e LNnT which are currently approved in supplemented formulas [13].

In fact, not every woman exhibits the same pattern of HMOs, as their synthesis shows interindividual variation, depending on different possible genotypes [14]. Supplemented formulas have been studied and tested in the past five years. Although not as effective, dynamic and tailored as mother’s breast milk, adequately supplemented formulas might compensate for either breastmilk unavailability or specific HMO deficiency. This is why, given the complexity of these molecules and the biological paths they belong to, future achievements in research are expected to benefit from a multidisciplinary approach, with the engagement of pediatricians, nutritionists, biologists and microbiologists [15]. Concerning “personalized medicine”, metabolomics appears to be the perfect strategy to understand/interpret one’s phenotype at the molecular level, leading to individualized care through the study of endogenous metabolites of biological fluids [16]. Metabolomics and “Omics-rooted” studies in general, with their high-throughput technology, are the perfect strategy to allow the precise characterization of human milk metabolome, glycobiome and lipidome [17]. Moreover, it offers great opportunities in understanding the relationship between human milk’s peculiar composition and infant outcomes in terms of growth and long-term health [18]. 

From the analysis of the literature available to date, even if there is a relevant amount of literature concerning human milk metabolome, and although the enthusiastic interest in the biological properties of human milk oligosaccharides, most of the evidence is based on in vitro studies, while minor attention has been given to metabolomic studies performed on cohorts of mother and infant pairs to investigate both biochemical characteristics and biological roles of these compounds. This review aims to provide an overview of human milk oligosaccharides as regards their biochemical, physiological, and clinical properties while also focusing on the application of metabolomics technology to characterize the structure and the functions of these fascinating metabolites. 

## 2. Human Milk Oligosaccharides: Structure and Composition

Human milk macronutrient percentage composition is approximately 0.8–0.9% protein, 4.5% fat, 7.1% carbohydrates, and 0.2% ash (minerals) [19]. Of the carbohydrate amount, 80% consists of lactose, while the remaining 20% consists of HMOs. Therefore, HMOs are the third most represented component in maternal milk [20,21]. Reported concentrations of HMOs result from the application of chromatography to separate HMOs from lactose [22,23], with estimated values of 5–15 g/L in mature milk and up to 20 g/L in colostrum [20,22]. 

HMOs are synthetized by glycosyltransferases, specific enzymes that variably combine five different monosaccharides into more than 150 different HMO structures with different roles [24]. These five monomers are: β-d-galactose (Gal), β-d-glucose (Glc), β-d-*N*-acetyglucosamine (GlcNAc), α-l-fucose (Fuc), and the sialic acid α-d-*N*-acetylneuraminic acid (Sia) [6]. The Lacto-*N*-biose unit is generated by a beta 1,3 linkage between galactose and *N-*acetylglucosamine. The *N-*acetyllactosamine unit is generated by alpha-1,4-linkage between galactose and *N*-acetylglucosamine. When the lactose moiety binds to a lacto-*N*-biose unit, elongation is no longer possible. Otherwise, when the lactose moiety is linked to a *N*-acetyllactosamine unit, the oligosaccharide can be further extended. Then, possible branching results from β1-6 linkage between two disaccharides [15,25].

Since oligosaccharides chains can be either fucosylated or sialylated, they are usually classified into three main categories: (a) neutral non-fucosylated HMOs (42–55%); (b) neutral fucosylated HMOs (35–50%); (c) HMOs containing sialic acid (12–14%) [26]. 

The most abundant HMOs include the 2′-fucosyllactose (2′-FL) which belongs to the fucosylated group. Lacto-*N*-tetraose (LNT) has been recently demonstrated to be the second most abundant HMO [27]. Others include the Lacto-*N*-neotetraose (LNnT), which belong to the neutral non-fucosylated [28]. 

The fucosyltransferases are specific enzymes responsible for specific oligosaccharide patterns synthetized by mothers based on their allelic expression of Secretor gene (α1-2-fucoslyltransferase, FUT2) and Lewis gene (α1-3/4-fucosyltransferase FUT3) [5,29,30]. The FUT2 synthetizes α1-2-fucosylated HMOs, especially 2′-FL [15,31].

Women who express at least one Secretor dominant allele (Se,se/Se,Se), are called “secretors” and synthetize high amounts of 2′-FL. Women who express two se recessive alleles (se,se), by contrast, are called “non-secretors”. Since they do not possess an active enzyme, their milk presents a significantly lower amount of 2′-FL and other alpha-1,2-fucosylated HMOs, if not undetectable [32,33]. This also explains why total HMO concentration in “non-secretor”‘s milk is 35–40% lower than in “secretor’s” [34].

The FUT3 synthetizes α-1,4-fucosilated HMOs (such as Lacto*-N*-fucopentaose, LNFP), which are abundant in the milk of women who express at least one Le dominant allele (Le,le/Le,Le): these women are called “Le positive”. While, those who express both FUT3 recessive alleles (le), are called “Le negative” and their milk lacks these α-1,4-fucosylated HMOs [35,36]. 

According to the genotype and the state of activation of FUT2 and FUT3, mothers can be divided into four groups:

Group 1: secretor, Lewis-positive, (Se, Le) (FUT2 active, FUT3 active);
Group 2: non-secretor, Lewis-positive, (se, Le) (FUT2 inactive, FUT3 active);
Group 3: secretor, Lewis-negative, (Se, le) (FUT2 active, FUT3 inactive);
Group 4: non-secretor, Lewis-negative, (se, le) (FUT2 inactive, FUT3 inactive) [37].

These four different genotypes and their related phenotypes show a variable geographical distribution: for example, up to 30% of certain African and Latin American populations show Lewis negative genotypes (Group 3 or 4), while the frequency of the same genotypes is about 4–6% in the white population [38]. Among Americans and Europeans, indeed, 80% of the population express the secretor phenotype [33], making Group 4 extremely rare. In contrast, in some African populations, over ~38% are non-secretors, with higher probabilities of mothers belonging to Group 4 [39]. 

The quantitative and qualitative patterns of HMOs depend on the different combination of allelic variants in one’s genotype, that results in either active or unactive form of FUT2 and FUT3. 

Moreover, the individual components of HMOs among different milk groups are modulated by the interaction and the competition between active enzymes. According to literature, indeed, active FUT2 and FUT3 compete for the same acceptor, Lacto-*N*-tetraose (LNT), which is preferentially converted by active FUT3 rather than FUT2. As a consequence, individuals belonging to Group 3 (FUT2 active, FUT3 inactive), show a higher concentration of 2′-FL and LNFP than individuals belonging to Group 1 (FUT2 active, FUT3 active), since they do not suffer from the FUT3 interference [40]. 

Moreover, among *Lewis positive* individuals, a higher concentration of LNFP-II and LNFP-III (Lacto*-N*-fucopentaose III), have been detected in non-secretor (Group 2) than in secretor (Group 1) individuals since there is lower competition for the acceptor LNT in the first than in the latter [40]. 

**Figure 1:**
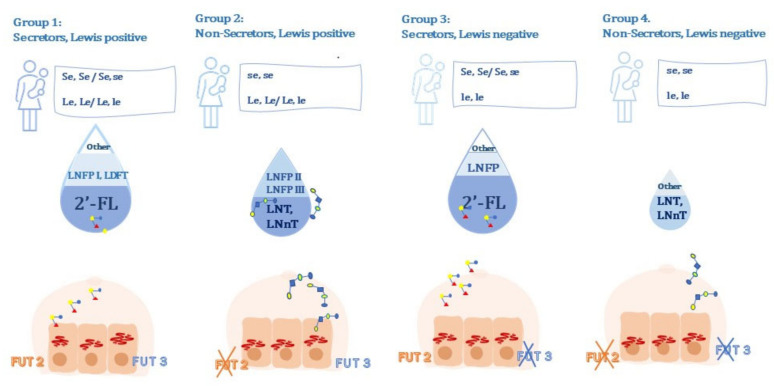
Correlation between genotype and phenotype in terms of mainly synthetized HMOs.

The main findings concerning HMO phenotype–genotype correlations are synthetized in Figure 1. 

Since alpha-1,2-fucosylated HMOs have been observed even in milk from non-secretor women, it has been supposed that other enzymes such as FUT1, might play a role in the synthesis of fucosylated-HMOs [41].

Moreover, other fucosylated HMOs, such as 3-FL (3-fucosyllactose) and LNFP-III, have been detected in milk from non-secretor or *Lewis negative* women [42,43], suggesting that there is a certain amount of HMOs synthetized by different fucosyltransferases (FUT 4,5,6,7 and/or 9), independently of the Secretor-Lewis genotype. 

All these structural and biochemical differences of HMOs seem to have a role in the possible several physiological functions, such as brain and intestinal development, exerted by this class of compounds. These functions will be discussed in the following paragraphs.

## 3. Human Milk Oligosaccharides: Biological Functions behind Physiology

### 3.1. Prebiotic Effect

The gastro-intestinal system of the newborn is immature both from a structural and immune point of view. Thus, the achievement of complete functionality is certainly a fundamental condition not only for the correct absorption of nutrients but also for protection from infections.

It is very important to establish a valid microbiota from the earliest stage of development, as this will affect future metabolic health [44].

Initially, the intestine of the newborn is colonized only by a few bacterial species: in the early stages, the species belonging to the *Firmicutes* and *Proteobacteria* phyla prevail, which will then be replaced by *Bacteroidetes* and *Actinobacteria*, thanks to which the species richness of the ecosystem is increased. However, among these, the genus *Bifidobacterium* soon becomes dominant. In fact, it can represent up to 90% of the microbiota of a breastfed baby while, in many formula-fed infants, they have been found in more modest quantities (about 50%), very similar to the concentrations of *Bacteroides* [45].

The dominance of this bacterial genus in the intestine of the breastfed infant is closely related to its optimal ability to metabolize the oligosaccharides of breast milk. This feature seems to be the result of an intense and selective evolutionary pressure of millions of years in order to better meet the survival needs of children [46]. However, this property is not exclusive to the *Bifidobacterium* genus but also belongs to some species of *Bacteroides*, *Enterococcus*, *Lactobacillus*, *Streptococcus* and *Clostridium* cluster IV/XIVa [45]. In fact, Borewicz et al. [47] showed that the degree of metabolization of oligosaccharides is closely related to the presence of the genera *Bifidobacterium*, *Bacteroides* and *Lactobacillus*.

However, among these, *Bifidobacteria* have evolved in close relationship with humans, developing unique characteristics, especially in infant-type species. In fact, in many strains of the *B. longum subspecies infantis*, they found a complex enzymatic kit that guarantees a very efficient metabolism of the oligosaccharides of breast milk [48]. On the contrary, other species, including *B. bifidum*, pour out intermediate metabolites at an extracellular level deriving from a partial digestion of oligosaccharides which, however, act as nourishment for other species, among which some belonging to the bifidobacterial ecosystem, thus favoring a real dominance of species [49]. However, differential utilization of intact HMO and HMO constituent monomers among other species, like Lactobacilli, is reported in the literature [50]. Moreover, the partially HMO digestion and the consequent accumulation of HMO constituent monomers observed by Thongaram et al. [50] suggests a possible cross-feeding capacity in the gut besides Bifidobacteria species.

In any case, the importance of these bacterial strains, capable of effectively metabolizing the oligosaccharides of breast milk, is due to the fact that they produce numerous essential substances for the human being, including some vitamins, protective factors against infections and short-chain fatty acids (SCFAs) [44,51,52]. Among these, the most important are: propionate, butyrate and acetate, which represent 90–95% of the SCFAs present in the colon [53]. They contribute to the energy needs of the human being, albeit minimally (10%), but above all to that of the colon cells (60–70%) [54]. They are also among the main culprits of lowering intestinal pH and increasing the production of mucins in the intestine with repercussions on bacterial adhesion. They also promote the increased activity of the adherent junctions (improved tight-junctions integrity) and are involved in the control of inflammation, through an action on the differentiation of T-regulatory cells [53]. This determines the modulation of the immune system and promotes the barrier function of the intestine. The biological effects of short-chain fatty acids also affect the central nervous system as they appear to be key molecules of the microbiota–gut–brain axis [55]. In addition, the digestion of breast milk oligosaccharides also favors the production of lactic acid, which is essential in helping to lower intestinal pH. This latter effect is crucial both in optimizing the absorption of nutrients and in favoring the antibacterial action of short-chain fatty acids which is mainly expressed through the entry of the non-ionized form, predominant at low pH, into bacterial cells [56].

### 3.2. Maturation of the Intestinal Barrier

The intestinal mucosa is a complex system, mainly composed of a single-cell epithelium and a lamina propria whose infiltration by numerous immunocompetent cells such as macrophages, dendritic cells and B and T lymphocytes is essential to ensure the barrier function. At the epithelium level, from crypts, multipotent stem cells continuously differentiate towards villi, into enterocytes, goblet cells, Paneth cells, enteroendocrine cells, M cells (microfold) and tuft cells [57]. The adherence of the barrier is guaranteed by the presence of adherent joints that control the paracellular permeability. On the other hand, the glycocalyx and mucus increase the effectiveness of the defensive system [57]. Data on the role of breast milk oligosaccharides on the maturation of this complex system are scarce [2]. Studies on animal models have shown an increase in the height [57,58] and area [59] of the villi as well as an increase in the depth of the crypts [60] but also an increase in enzymatic activity at the border brush [59]. These results, if confirmed in humans, support the hypothesis of the ability of oligosaccharides to optimize intestinal absorption. Kuntz et al. [61] showed that the oligosaccharides of breast milk inhibit the cell growth of intestinal cell lines in vitro, interfering with the progression of the cell cycle through the induction of differentiation and/or influencing apoptosis. These findings are important since cell differentiation is a fundamental process in the development and maturation of digestive systems and absorption processes. In addition, it seems that this modulation is due to a specific action both on particular cell cycle regulatory genes and on MAPK (mitogen-activated protein kinase) dependent signaling. Hester et al. [62] also confirmed an anti-proliferative action of breast milk oligosaccharides on the growth of epithelial cell lines. Further confirmation is the study by Holscher et al. [63] which showed an anti-proliferative effect for three breast milk oligosaccharides (2′-FL, 6′-SL and LNnT) in a model of epithelial cells of the crypto-villus axis. Nevertheless, the effects on differentiation, digestive processes and barrier function differed between the tested oligosaccharides, thus suggesting different specific actions for each individual oligosaccharide under consideration. Further research on the induction of differentiation of epithelial cells through the individual administration or in a combination of particular mixtures of breast milk oligosaccharides has shown an anti-proliferative effect associated with an increase in epithelial differentiation. It was also shown that the observed effects were additive but no specific combination was better than others [64]. Given the importance of the glycocalyx at the level of the intestinal epithelium for microbial colonization, Kong et al. [65], for the first time, studied its development in correlation with the oligosaccharides of breast milk. The results showed that two oligosaccharides in breast milk, 2′-FL and 3-FL, directly influence the development of epithelial cell lines by stimulating the development of the glycocalyx.

As regards mucus, another crucial component of the intestinal barrier system, Cheng et al. [66] has shown that some oligosaccharides in breast milk increase its production through a direct modulation of goblet cells.

Finally, with regard to intestinal permeability, a study on animal models has highlighted possible sex-specific effects, noting a decrease in tight junction-mediated intestinal permeability in specimens of female rats [67].

### 3.3. Brain Development

The high growth rate of the brain during the first six months of the newborn’s life determines a high demand for target nutrients for neuro-cognitive development [59]. This period coincides with the timing recommended by the WHO for exclusive breastfeeding and the related benefits have been widely demonstrated. In fact, it was found that breastfed newborns have better “intelligence test scores” during childhood, with repercussions also in adulthood [68]. There is therefore a window of opportunity to support proper brain maturation in the early stages of development and in this context, nutrition is known to have a significant impact [69]. As reported by Fleming et al. [69], among the possible mechanisms underlying the correlation between the brain and the intestine, there are alterations of the neurotransmitters of the central nervous system, changes in the microbiota with the related metabolites and direct vagal mediation. In this regard, various HMOs were analyzed including 2′-FL and those containing sialic acid. Preliminary studies on animals have shown that the administration of 2′-FL influences the cognitive domain and increases memory and learning skills in rats [70] with an effect that seems to depend on the integrity of the vagal nerve. These data further support the fact that the gut –brain axis plays a key role in mediating the positive effects of 2′-FL [71]. Fleming et al. [69] demonstrated how, in animals, breast milk and bovine oligosaccharides have distinct effects on both brain structure and cognitive development. These results therefore suggest that the mechanisms underlying the influence of the different oligosaccharides on neuro-development vary according to the analyzed type [69]. The first study on the effects of 2′-FL on humans was performed on 50 mother–child pairs by Berger et al. [68]. They showed that a greater frequency of breastfeeding at one month of life contributed to the cognitive development of children through greater exposure to 2′-FL compared to other oligosaccharides. However, the effects of 2′-FL at 6 months of life were not significant, suggesting that early exposure to 2′-FL probably represents a critical time window for the positive influence on the cognitive development of the newborn. The “sialylated” component of oligosaccharides (sialylated HMOs) has also been found to be very important for what concerns neurological development, as the cell membranes of neurons are the richest in sialic acid. In fact, it is a component of gangliosides, glycosphingolipids with a hydrophilic head consisting of a sialylated oligosaccharide, which is therefore involved in various aspects of neuro-development such as synaptic transmission, memory, cognitive and learning abilities [72]. Furthermore, in the brain, the concentration of gangliosides increases three times from the tenth week of gestation to the fifth year of life [73], supporting the importance of this molecule during early childhood. However, even in this area, the main information comes from animal studies. Among these, Wang et al. [74] showed that the supplementation of sialic acid in pigs has increased learning abilities by also increasing the expression of specific related genes. In other animal experiments, it emerged that two oligosaccharides containing sialic acid normally present in breast milk, 3’-SL and 6′-SL, diminish the stressor-induced anxiety-like behavior in rat pups [55]. Oliveros et al. [75] detected the positive effects of taking 6′-SL in early developmental stages on the memory and learning abilities of animals in later developmental stages as well. Additional benefits following sialyllactose supplementation have been observed in the cognitive development of preterm pig pups [76].

### 3.4. Development of the Immune System

HMOs provide immunomodulation through their interaction with pathogens, cell receptors and immune cells at a systemic level but also through their influence on gut microbiota. Since hydrolysis of HMOs, by digestive enzymes of the small intestine of humans, in the upper gastrointestinal tract is prevented due to the lack of sialidases and fucosidases [77,78], these compounds reach the large intestine undigested. Here, a small amount (1%) is absorbed into systemic circulation, with detected systemic levels within 10–100 mg/mL, then reaches the peripheral blood and interacts with immune cells [79]. Most HMOs, instead, are digested by the infant gut microbiota thus shaping its composition and promoting the growth of favorable bacteria [78,80]. Different cells of the human immune system display different types of glycan-binding proteins, called lectins, which act as receptors for antigenic portions of carbohydrates. Therefore, specific HMOs can bind to specific lectins expressed on immune cells such as Galectins [81], Sialic acid-binding immunoglobulin-like lectins (Siglecs) [82] and Selectins [15].

Galectins are highly conserved lectins expressed by antigen-presenting cells (APC), granulocytes, T cells, intestinal epithelial cells and pathogens [83]. These β-galactoside-binding proteins exert different roles in both innate and adaptive immune responses, recognizing glycan structures on immune cells, influencing signaling pathways or binding with glycans displayed by pathogens [84]. Four galectins (Galectin-1, -3, -7, and -9) are able to bind different HMOs with specific affinities. In particular, they show moderate-to-high affinity to 2′-FL, the most common oligosaccharide in human milk [85]. Immunomodulation might result from the interaction of galectins and HMOs, as suggested by the immune-modulatory and anti-inflammatory effect of 2′-FL in vitro. In fact, this compound reduces the activation of monocytes and modulates the expression of CD14 in human enterocytes [86]. As well as galectins, Siglecs are expressed by immune cells and they exert their roles in immune responses too, as they interact with eosinophils and mast cells. These lectins are reported to bind mainly sialylated HMOs, which, in turn, might have a role concerning allergy prevention [87].

Moreover, sialylated HMOs specifically interact with Selectins, which are expressed by endothelium cells and regulate lymphocyte rolling at sites of inflammation. The binding between these HMOs and Selectins affects interaction among leukocyte and platelets or endothelium cells [88].

Several studies have shown that HMOs achieve their immunological effects through Toll-Like Receptor (TLR) signaling [89].

Cheng et al. [90] demonstrated that the immunomodulatory effects of HMOs via TLRs are structure-dependent, as they analyzed the interaction of 2′FL, 3-FL, 6′SL, Lacto-*N*-triose II (LNT-II), and LNnT with specific TLRs in vivo. The authors revealed that while LNT-II had TLR stimulating effects, the 2′-FL, 6′SL, and LNnT had inhibiting effects on TLR5 and 7. Moreover, 3-FL had both stimulating effects on TLR and inhibiting effects on TLR5, 7, and 8.

Moreover, more HMOs may have a specific impact on immuno-modulation based on their specific biochemical structure. Namely, LNFP-III has been shown to act as an innate Th2 promoter in vivo, for it stimulates maturation of murine dendritic cell 2 phenotypes and the release of IL-4 (Interleukin 4) and IFN-γ (Interferon-gamma)[91].

Promising results emerge from the investigation of Goehring et al. [92], who demonstrated that infants fed a 2′-FL supplemented formula exhibit lower concentration of inflammatory cytokines (IL-1ra, IL-1α, IL-1β, IL-6, and TNF-α, tumor necrosis factor-alpha) compared to infants fed the control formula.

Doherty and colleagues [11], systematically reviewed 10 articles from 6 different studies and found out that the abundance of HMOs was associated with reduced immune-mediated diseases and infection, such as cow’s milk allergy, gastroenteritis and respiratory tract infections, reduced human immunodeficiency virus (HIV) transmission and lower mortality among exposed infants. Therefore, they suggest that the impact and the benefits of HMOs may result in early programming of the immune system itself.

### 3.5. Protection against Allergies

The prebiotic effects and the immunological programming provided by HMOs also affect individual susceptibility to allergies, through strictly connected mechanisms. Firstly, healthy microbiota and microbiome have crucial immunological benefits as they reduce the risk of allergic disorders [93] thanks to their production of SCFAs, such as butyrate and propionate that exhibit anti-inflammatory and anti-allergic properties. Indeed, Walsh and colleagues [94] showed that these compounds protect against the risk of asthma and food allergy. These findings were supported by Trompette et al. [95], who demonstrated that low gut microbial SCFA production in mice exposed to house dust correlates with higher levels of IgE (Immunoglobulin E), goblet cell hyperplasia and increased mucus in the airways.

As suggested by different authors, species such as *B. longum* apparently play a protective role against asthma and allergic dermatitis [96,97].

Secondly, HMOs directly interact with the immune system as explained in the precedent paragraph [15,81,92]. Hence, a protective role of HMOs against allergies has been inferred based on these mechanisms and several in vitro studies have supported this hypothesis. In a murine food allergy model, bone-marrow-derived mast cells exposed to 6′-FL displayed an attenuated IgE-dependent mast cell degranulation; treatment with 2′-FL and 6′-SL also reduced food allergy symptoms in the mentioned animal models [98]. Treatment with synthetic fucosylated HMOs inhibited T_H_ cell expansion in piglets, suggesting a potential role in the attenuation of allergic inflammatory reactions [99]. Recent investigation on the potential claims of HMOs has suggested a correlation between the reduction in both allergies and neonatal infections with the addition of prebiotics and oligosaccharides in the diet of the neonates. Zehra et al. [100] demonstrated that 2′-FL and 6′-sialyllactose (6′-SL) stimulate TNF-α and prostaglandins E (PG-Es), which are protective factors against allergies.

### 3.6. Protection from Infections

Researchers have been focusing on the human milk components that may act as anti-infective and immunomodulating factors. Among these compounds, HMOs play a determining role, as they exert a prebiotic effect promoting the development of a favorable microbiota that competes with harmful bacteria [101], and they might directly prevent pathogen adhesion and colonization [41]. Microbial adherence to epithelial human cells depends on the binding of pathogen receptors to glycans exposed on the human cell surface. In this context HMOs and glycoconjugates of human milk, like glycoproteins and glycolipids, act a crucial role, indeed they synergistically participate in the inhibition of pathogen adhesion. Due to their structural similarities with these epithelial surface glycans, HMOs and glycoconjugates can participate in two different ways: they can be recognized and bound by bacterial lectin receptors or by newborn epithelial cells, in both cases thus preventing pathogen colonization [102,103]. The team led by Craft and Townsend applied plate-based assays to demonstrate that purified HMOs are able to inhibit bacterial growth of *S. Agalactiae* and *A. Baumannii* while they also possess antibiofilm activity against *S. Aureus* and *S. Agalactiae*. Concerning *S. Agalactiae*, in particular, LNT-II showed the strongest antimicrobial activity compared to other single HMOs, with an average growth reduction of 54%. Heterogeneous HMO extract, though, were most effective than single HMOs, with average growth reduction of 82% [103]. Indeed, not only do HMOs act as prebiotics, but they can also be considered as antiadhesive antimicrobial compounds. Sialylated and fucosylated milk oligosaccharides reduce the incidence and the severity of gastrointestinal infections as they inhibit interactions among pathogens and host receptors as demonstrated for *Rotavirus* [104,105], *Norovirus* [106], *Campylobacter* and *Escherichia Coli* [107]. Moreover, higher concentrations of alpha-1,2-linked fucosyl-oligosaccharides in mother’s milk correlate with a lower risk of diarrhea in breastfed infants [108].

Concerning protection against respiratory tract infections, Puccio and colleagues [109] have demonstrated how the supplementation of a regular formula with 2′FL and LNnT correlate with fewer parent-reported episodes of bronchitis and medication use. Kwon et al. [110], instead, found that HMOs enhance the innate immune response to influenza viruses, as they interfere with hemagglutination.

The bioactive role of HMOs could be responsible for the uncommon HIV transmission via breastfeeding, as only 10–15% of breastfed infants acquire the virus from their HIV-infected mothers. Bode et al. [15] demonstrated that higher concentrations of HMOs (except for 3′-SL) correlate with a lower risk of postnatal HIV transmission in breastfed infants since they might interfere with the binding of HIV to dendritic cell-specific intercellular adhesion molecules.

### 3.7. Protection against Necrotizing Enterocolitis

Necrotizing Enterocolitis (NEC) is a life-threatening gastrointestinal condition, occurring mainly in preterm newborns with very low birth weight, due to their mucosal and microbiota immaturity. The incidence of this devastating disease is 6–10 times lower in infants who receive exclusively their mother’s milk [111]. Based on the evidence that preterm breastfed infants show lower morbidity and mortality due to NEC compared to formula-fed infants [112], a protective role of HMOs against NEC has been postulated already in 1990 [111] and supported by a consistent amount of literature ([112], and reference therein). Indeed, infant formula lacks complex glycans that are abundant in human milk. Moreover, HMOs act as protective factors against microbial dysbiosis, intestinal and immune system immaturity, which are recognized as the main etiological factors behind NEC [113].

Moreover, breast milk of mothers who delivered prematurely shows high levels of HMOs [114], suggesting an active regulation of these compounds, which adapt to the immunological needs of infants as well to their nutritional necessities.

Initially, this hypothesis was reinforced by results derived from in vitro and animal studies performed mainly on rat models of NEC. In an interesting study from Jantscher et al. [115], rats who received a special formula containing extracted HMOs, had significantly better outcomes compared to rats who received a controlled formula without HMOs. Indeed, rats from the first group showed lower pathology scores presented lower levels of necrosis and fewer hemorrhagic lesions. Subsequently, the same authors demonstrated that among different HMOs, disialyllacto-*N*-tetraose (DSLNT) is the one responsible for protection against NEC in rat models, at physiological concentration.

At a molecular level, the role of HMOs has been elucidated by Wu and colleagues [116], who showed that formula supplemented with HMOs lead to increased levels of mucin in rat NEC models, reduced intestinal permeability and attachment of enteric pathogens.

Eventually, the protective role of HMOs was confirmed by human studies, too. Results from a multi-centered cohort analysis performed on 200 mothers who delivered low birth weight infants, suggest that a higher concentration of DSLNT in breastmilk correlates with a lower risk of NEC in low-birth-weight newborns [117,118].

Recent interesting findings suggest that HMOs may prevent injury of the intestinal mucosa by modulating the expression of TLR4 on epithelial cells. TLR4 plays a critical role in the pathogenesis of NEC, since its activation is responsible for the exaggerated inflammatory response that results in increased intestinal permeability. HMO treatment in mice NEC models downregulated TLR4 expression on intestinal cells, thus reducing inflammatory damage and promoting crypt cells turnover [119]. Moreover, among HMOs, sialylated oligosaccharides at their physiological levels, have been reported to improve survival in rat NEC models, by suppressing TLR4-mediated inflammation, while also downregulating the NLRP3 inflammasome [120]. Namely, supplementation of formula with 2′-FL and 6′-SL inhibits TLR4-mediated inflammation in mice and piglet NEC models, resulting in reduced apoptosis, inflammation, weight loss, and less severe histological appearance compared to mice and piglets fed control formula [121].

In conclusion, the beneficial effects of HMOs against NEC may be interpreted as a comprehensive combination of all the previously reported effects. Indeed, HMOs exert their protective role against each of the risk factors contributing to the development of NEC. First, they act as prebiotic, supporting a favorable microbiome to prevent pathogen growth. Second, they contribute to the integrity of the intestinal mucosa. Third, they act as antimicrobials, preventing pathogen adhesion to epithelial cells. At last, they modulate the immune and response, thus limiting the magnified inflammation occurring during NEC.

**Figure 2:**
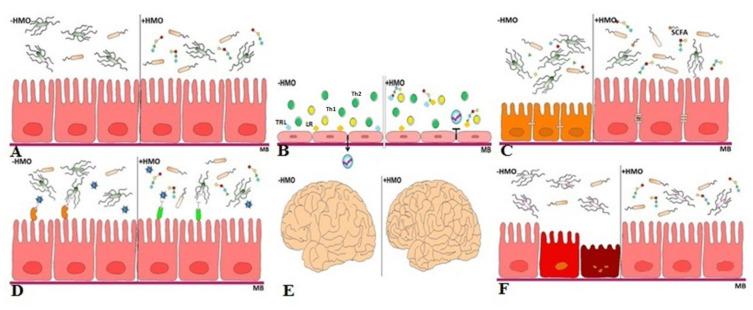
Effects of HMOs: (**A**) Prebiotic Effect: the bacteria that are able to metabolize the HMOs or their constituent monomers (cross-feeding) become commensal, while those that do not, are eliminated; (**B**) Development of the immune system: HMOs provide immunomodulation through their interaction with specific human immune cells; (**C**) Maturation of the intestinal barrier: HMOs promote the maturation of the intestinal barrier through different mechanisms optimizing intestinal absorption; (**D**) Protection against infections: two ways of participation of HMOs in inhibition of pathogen adhesion are possible: they can acts ligands for lectin-receptors of bacteria and/or viruses as well as for leptin receptors of newborns’ epithelial cells; (**E**) Brain development: sialic acid, delivered with mother’s milk, may support the brain development; (**F**) Protection against NEC: a comprehensive combination of all the previously reported effects may be beneficial against NEC.

The specific biological effects of HMOs are summarized in Figure 2.

## 4. Metabolomics and Human Milk Oligosaccarides 

### 4.1. Methods: Inclusion and Exclusion Criteria

The authors decided to include Pubmed, Researchgate and Scopus as source databases. The research covered the time period from 2012 to 2021 and it was conducted at the beginning of 2021. Concerning research criteria, articles and papers with the following words within the title, the abstract and the keywords were included: (“Human Milk Oligosaccharides” OR “HMOs” OR Human Milk Glycobiome) AND (Metabolomics OR H-NMR OR HPLC OR Chromatography); animal and in vitro studies were excluded.

### 4.2. Metabolomics and Human Milk Oligosaccharides: Review of the Literature’s Results

In the past 20 years, metabolomics appears to have become the prevailing strategy in the study of different classes of nutrients, due to its high-throughput technologies. *High-Performance Liquid Chromatography* (*HPLC*), mass spectrometry (MS), Proton Nuclear Magnetic Resonance (^1^H-NMR) and nano-Liquid Chromatography chip Time-Of-Flight Mass Spectrometry (nano-LC chip-TOF MS), allow the characterization of different low-molecular-weight metabolites in human biofluids, including HMO structures. Different authors have stressed the potential of metabolomics-based analysis to comprehensively investigate human milk composition, especially in terms of carbohydrates and fatty acid moieties, while also achieving a better understanding of infant health and nutrition [122,123,124].

The research produced 19 results, listed in Table 1.

According to our research, the first study was performed by Newburg et al. [106], in 2004. The authors described the HMO patterns expression related to different Lewis’ phenotypes, while also investigated the protective effects of different genotypes-phenotypes against *E. coli* heat-stable enterotoxin. Indeed, they found LNF-I and 2′FL to be the main HMOs in milk from Le^a−b−^ mothers, and LNF-II, LNF-III, 3-FL in Le^a−b+^ mothers. Additionally, they demonstrated that infants consuming milk with lower concentrations of alpha-1,2-linked fucosyloligosaccharides (2′-FL) more frequently developed moderate to severe diarrhea compared to infants whose milk was rich in this compound. Since milk of non-secretor mothers lacked 2′-FL, they suggested Secretor genotypes might have a protective role against diarrhea due to *Escherichia coli* heat-stable enterotoxin [108]. These results have been supported by Thurl et al. [14], who confirmed that the pattern of HMOs synthetized by mothers, correlates to their phenotype and genotype, for it depends on the active state of the FUT2 and FUT3. Additionally, the authors were able to demonstrate that HMO concentration varies during lactation, as it reaches its peak during the first week postpartum and then decreases about 50% at three months [14]. Variation of HMOs during lactation was also investigated by Gabrielli et al. [114], who observed a reduction in total HMOs during the first month in all groups. Nevertheless, different groups displayed different trends: in fact, a slower decrease was observed in Group 1, in which higher total HMO concentration in early phases of lactation was also described.

Concerning the composition of glycobiome, Totten and colleagues [125] focused their attention on the different HMOs displayed by mothers presenting different Secretor status and Lewis’s phenotypes. They found that the most significant differences concerned the amount of fucosylated HMOs, that were prevalent in secretor individuals. Thus, they suggested that HMO patterns depend on the secretor status rather than on the Lewis’s phenotype.

In 2012, the research team led by Bode [15] investigated the role of breast milk and HMOs in HIV transmission. They found that breast milk of mothers who did not transmit the HIV virus to their offspring showed a higher content of non-3′-sialyllactose (3′-SL). Thanks to metabolomics it was possible to investigate and support the hypothesis that HMOs and breastmilk could be protective against HIV transmission.

Different authors were able to differentiate breast milk on the basis of the secretor and non-secretor phenotype, regardless of the different metabolomics technology applied. Their studies demonstrated the abundance of α1-2 fucosylated HMOs (2′-FL, LNFP-I) in secretor’s milk and the absence of α1-2 fucosylated HMOs in non-secretor’s milk [39,40,123,126,127,133].

Moreover, Sammuel et al. also correlated higher concentrations of LNT and LNnT in the milk of overweight mothers compared to those observed in milk from mothers with normal pre-pregnancy BMI (ppBMI) [39]. This aspect was also recently investigated by Saben et al. [135], who demonstrated that HMO composition differs among mothers with normal weight, overweight or obesity.

Spevacek et al. [129], in 2015, through the ^1^HNMR metabolomics analysis of 15 milk samples of women who delivered at term and 13 milk samples of women who delivered preterm, found that HMO quantity varies according to the lactation stage and the gestational age of neonates. They detected the following HMOs: 2′-FL, 3-FL, 3′-galactosyllactose (3′-GL), Lactodifucotetraose (LDFT), LNFP-I, LNFP-II, LNFP-III, LNnT, LNT, 3′-SL, 6′-SL. Lactose, 3-FL and glucose increased with the maturation of milk: lactose increased from 180 ± 12.8 mmol/L at birth to 180 ± 14.6 mmol/L at 28 days in term neonates and from 150 ± 16.5 mmol/L at birth to 170 ± 12.6 mmol/L at 28 days, in preterm neonates; 3-FL increased from 0.91 ± 1.05 mmol/L at birth to 1.57 ± 1.34 mmol/L at 28 days in term neonates and from 0.99 ± 0.77 mmol/Lat birth to 1.98 ± 1.39 mmol/L at 28 days, in preterm neonates. While, there was a reduction over the same period (from birth to 28 days) concerning 2′-FL (from 5.43 ± 4.55 mmol/L to 3.59 ± 2.83 mmol/L in term neonates and from 4.96 ± 4.7 mmol/L to 2.32 ± 2.54 mmol/L in preterm ones), 3′-GL (from 0.83 ± 0.26 to 0.51 ± 0.15 mmol/L in term neonates and from 1.00 ± 0.29 mmol/L to 0.82 ± 0.40 mmol/L in pre-term ones), LNFP-III (from 0.42 ± 0.22 mmol/L to 0.26 ± 0.09 mmol/L in term neonates). Moreover, concerning LFNP III and LNnT in preterm, their concentrations did not show a significant variation during the first 28 days of breastfeeding (0.28 mmol/L vs. 0.27 mmol/L and 0.23 mmol/L vs. 0.20 mmol/L, respectively). Another study performed by Sundelkilde et al. [130] compared preterm milk vs. at term milk over a longer course of lactation, 45 samples of breast milk in total: 30 at term and 15 preterm. The fucosylated HMOs and other monosaccharidic compounds (fucose, *N*-acetylneuramic acid and *N*-acteylglucosammine) were found to be more elevated in the colostrum while their levels reduced in mature milk. Some HMOs were more elevated in the preterm newborn, in particular the LNT, LNDFH-I, 3′-SL, 6′-SL, and other HMO components, including fucose, *N*-acetylglucosammine and *N*-acetylneuraminic acid [130]. Even the charged HMO (represented by il 3′-SL e il 6′-SL) varied significantly according to the stage of milk maturation (colostrum: 20% at term, 39% in preterm; transition milk: 20% at term, 32% in preterm; mature milk: 24% at term, 53% in preterm). In the sample population, 10 out of 15 mothers that delivered at term and 10 out 13 that delivered before the 37 weeks presented the secretor phenotype. Furthermore, the authors speculated that metabolomics of breast milk concerning the HMO content could be applied in clinical practice in the future since they hypothesized that the HMOs found in preterm milk could be one of the causes of the low incidence of NEC in breastfed preterm.

Interesting results were published by Alderete and colleagues [128] regarding the correlation between HMO composition and infant growth and obesity. The authors enrolled 31 breastfeeding mothers–infant pairs and investigated the effects of different HMOs and infant growth at 6 months of age. They found that LNFP-II and DSLNT correlate with greater fat mass, while LNFP-I inversely correlates with infant weight and fat mass.

Charbonneau and colleagues [131], in 2016, characterized the HMO composition of two cohorts of Malawian mothers whose 6-months infants were either healthy or severely stunted. Cohort 1 included 88 mothers, while Cohort 2 included 215 mothers. In both cohorts, secretor mothers (n1 = 69; n2 = 155) exhibit higher fucosylated HMO amounts in their milk, compared to non-secretor mothers (n1 = 19; n2 = 60). Interestingly, while among secretor mothers there were no differences in HMO concentrations whether they had healthy or stunted infants, among non-secretor mothers, those with severely stunted infants presented significantly lower concentrations of fucosylated and sialylated HMOs compared to mothers of a healthy infant. Based on this evidence, the authors supposed that milk from non-secretor Malawian mothers might be less supportive of infant growth because of an ineffective compensation of fucosylated HMO deficiency [129]. 

The study performed by Mc Guire et al. [132], tested the hypothesis that the geographic location and ethnicity of mothers may exert some effect on HMO content of milk. The concentration of 3′-fucosylalctose in the milk of Swedish mothers has resulted to be four-fold higher than that of Gambian mothers, while the disialyllact-N-tetraose is reduced. The changes are not only based on ethnicity but there are differences in the same nation as well. In the Gambian cohort, the quantity of LNnT changes significantly between the rural and the urban area. 6′-SL, LSTc and FLNH are more elevated in Ethiopia in the urban area rather than the rural area. However, the modifications occur not only according to the genetics profile but also epigenetic may notably influence the oligosaccharidic expression. Maternal weight and BMI are positively correlated to the 2′sialyllactose and FLNH, while it results to be inversely correlated to the LNnT and the DSLNT. The post-partum period is inversely proportional to different HMOs including 6′-sialyllactose, LNFP-III, LSTc, latco-N-hexaose, DSLNT [132].

For what concerns HMOs, they change overall the proportion according to the mothers’ nationality. Several authors described this phenomenon in literature. For instance, Gomez et al. [134] in 2018 performed a study in which they observed that in Finland and Spain there is an abundance of LNFP-I and 2′-FL while 3-FL e LNFP-III is more abundant in South Africa and China. They also mentioned that the effect of C-sections on breast milk composition varies according to the nationality of mothers as well. Furthermore, the authors highlighted the role of the analysis of breast milk in different parts of the world since different HMO production may have different effects on the health and the development of neonates.

Recently, Wang et al. [39] have performed a study on a cohort of 10 healthy breast-feeding mothers. First, they have confirmed previously reported results in terms of HMO composition related to different genotypes. Second, they investigated whether any difference in the non-HMO milk metabolome exists among different genotypes. Interestingly, they found that significant differences exist among non-secretor/Lewis negative individuals and others. Thus, they suggested that the mammary gland metabolome is widely influenced by the inactivity of FUT2 and FUT3, since mothers with a Se–Le– phenotype show different non-HMO metabolome compared to other phenotypes.

In conclusion, metabolomics was useful to investigate the protective role of HMOs against HIV transmission [6], to characterize specific patterns of HMOs related to genotypes [39,40,123,126,127,133] and to support the hypothesis that HMO content in breastmilk varies according to the gestational age and the stage of lactation [14,114,129,130], and that there are differences in HMO production and secretion according to the location and the ethnicity of the women [132,134]. 

## 5. Human Milk Oligosaccharides Interindividual Variability: Is It Always about the Genes?

While the maternal genetic factors behind different HMO patterns have been widely investigated in the literature, there are few studies concerning the role of other factors such as maternal health, nutrition, and environmental influences. The impact of BMI on the glycobiome has been postulated by different authors. According to the recent review by Biddulph and colleagues [136], an influence of body composition and maternal dietary intake on HMO biosynthesis can be cautiously inferred. Apparently, indeed, mothers with higher pre-pregnancy BMI display different HMO patterns (richer in 3′-SL, 6′-GL and lower in LNT) compared to mothers with regular pre-pregnancy BMI [40,137]. Recently, Saben and colleagues [135] have supported the hypothesis of a pathogenetic connection among HMOs and maternal body composition and nutritional status. They performed HMO analysis of 194 breastfeeding women that were previously enrolled in two longitudinal studies and that presented no pre-existing ongoing medical condition, except for overweight or obesity. Indeed, women were classified based upon their BMI: in Normal Weight (*n* = 68), Overweight (*n* = 51), or Obese (*n* = 75). They explored the relationship between HMOs and maternal BMI, but also between HMO composition and infant growth until 6 months. According to their findings, maternal BMI shows a linear direct correlation with LNnT, 3-FL, and 6′-SL concentrations. Moreover, 3-FL and 6′-SL are also positively correlated with infant fat mass, supporting the idea that HMO variations due to maternal obesity might affect infant adiposity consequently. These findings suggest a potential predictive role for infant growth, with promising future opportunities in nutritional programming. Concerning sialylated HMO concentrations (DSLNT, DSLNH, and FDSLNH), instead, these were negatively associated with maternal BMI, resulting in a lower concentration of these compounds in milk from obese mothers compared to overweight and normal-weight ones [135].

Azad and colleagues [138] demonstrated that other environmental factors such as season and geographic location studied within the same country (Edmonton, Vancouver, Manitoba–Canada) apparently correlate with different concentrations of LNnT, 2′-FL and LNT.

## 6. Formula Milk and HMOs Supplementation: Where Next?

The application of bioengineering to induce microbial fermentation has allowed the production of synthetic analogues of 2′-FL and LNnT to supplement regular infant formulas [13]. Tolerance and safety of HMO-supplemented formulas have been tested by different clinical trials which demonstrated that the growth of supplemented-formula-fed infants is similar to that of breastfed infants [109,139]. Puccio et al. [109] also reported that parents of infants fed the 2′-FL and LNnT supplemented formula referred to lower episodes of bronchitis compared to those fed regular formulas without HMOs. Moreover, infants fed 2′-FL supplemented formula also exhibit lower inflammatory cytokine levels, similarly to breastfed infants [92] those fed the formula with 2′FL and LNnT showed a breastfed-like gut microbiota [140]. Apparently, supplement formulas not only allow adequate growth, but also mimic physiological properties of natural HMOs, including immune-modulating and prebiotic effects.

There might be further future applications of synthetic HMOs, as supported by in vitro study by Castillo-Courtade and colleagues [98], who demonstrated a beneficial effect of oral treatment with 2′-FL and 6′-SL in a mouse model of food allergy. Oral supplementation, indeed, reduced diarrhea and hypothermia in mice and also resulted in reduced passive cutaneous anaphylaxis response. Inhibition of mast cell degranulation was also demonstrated in vivo by the authors. Future achievements in this field might come from the investigation of the therapeutic potential of HMOs and their nutritional benefits in infants and children, too.

## 7. Conclusions

Breast milk, with its complex and dynamic composition, has deep positive impacts on the health and development of newborns and infants, with great long-term benefits for children and adults. In particular, in this paper, we discussed the several physiological and protective roles of HMOs, such as brain and intestinal development or protection from infections. This is one of the miracles of human milk: a single class of molecules has such a great impact on the development of the infant. In this context, metabolomics, with its ability to detect the metabolites in biological samples, offers multiple applications both in clinics and research. Thus, we reviewed the metabolomics data concerning HMOs present in the literature up to the beginning of 2021. Metabolomics was able to distinguish the different phenotypes related to specific patterns of HMOs and allowed the investigators to understand that the genotype is not the only factor that determines the production of HMOs. In fact, there are other modifiable and unmodifiable factors, such as ethnicity, geographic location, maternal diet and season, that influence HMO production from a qualitative and a quantitative point of view. Furthermore, metabolomics studies confirmed the hypothesis of the dynamic composition of breast milk and thus HMOs, meaning that they vary according to the gestational age of newborns, stage of lactation and even nutritional needs of each infant. Given the importance of these compounds and their ability to influence the health of a neonate, the knowledge of a mother’s phenotype is fundamental for clinicians. For instance, it would be very important to know whether a particular human milk oligosaccharide that is thought to be protective against NEC is present in the milk of a mother or not, possibly supporting infants with adequate supplementation. We believe that in the future, thanks to the progress of metabolomics, a dipstick-based real-time profiling of breast-milk HMOs will be possible. We could cautiously hypothesize that, as research progresses into a more detailed and comprehensive knowledge of HMOs and their biological activity, we will be able to achieve a specific supplementation of mother’s milk or infant formula, guaranteeing an optimal nutritional regimen for each newborn. Personalized medicine and personalized nutrition are the keywords driving the application of metabolomics in neonatology, for what we are able to do in the present to protect a newborn will affect the future adult as a consequence.

## Figures and Tables

**Table 1:** Metabolomics studies and oligosaccharides present in breast milk.

Authors, Year	Milk Sample	Technique	Results	Clinical Significance
Newburg et al. [108], 2004	93 healthy Mexican breast-feeding mothers (67 Le^a−b+^, 24 Le^a−b−^, 2 Le^a+b−^)	HPLC	↑ LNF-I and 2′-FL in milk from Le^a−b−^ mothers, ↑ LNF-II, LNF-III, 3-FL Le^a−b+^ mothers ↓ 2-linked fucosyloligosaccharides in milk consumed by infants with diharrea due to *E. coli* heat-stable enterotoxin (milk of non secretors mothers)	Different Lewis phenotypes correlate to different patterns of HMOs Non secretor genotype, results in lower amounts of 2′-FL and higher risk of diarrhea due to *E. coli* heat-stable enterotoxin
Thurl et al. [14], 2010	30 healthy Caucasian breast-feeding mothers (22 Le^a−b+^, 3 Le^a−b−^, 5 Le^a+b−^)	HPAEC	↑ LNFP-II, LNDFH-II, 3-FL in in milk from Le^a−b+^ mothers No α1,4-fucosyloligosaccharides in milk from Le^a−b−^ mothers No α1,2-fucosyl-oligosaccharides in milk from Le^a+b−^ (Non-secretors) mothers Highest levels of LNT and LNH reached at 1 week postpartum; 50% decrease by 3 months postpartum	Different Lewis phenotypes correlates to different patterns of HMOs HMO concentrations vary during lactation, reaching their highest concentration during the first week of life
Gabrielli et al. [114], 2011	63 breast-feeding mothers who delivered preterm (25–30 w)	HPAEC	Higher total HMO concentration in milk from Group 1; reduction in total HMOs during the first month in all groups; 2′-FL, DFLNH, and LNFP main HMOs in Group 1; DFLNH, LNFP-II, and 3-FL main HMOs in Group 2; 2′-FL and LNFP-I main HMOs in Group 3; MFLNH-II and LNFP-III main HMOs in Group 4	Different genotypes correlate to different patterns of HMOs (Group 1–4) HMO concentrations vary during lactation, with concentration decreasing more slowly in Group 1
Totten et al. [125], 2012	60 Gambian breastfeeding mothers (27 secretors Le^a−b+^, 6 non secretors Le^a+b-^, 27 secretors Le^a−b-^, 10 non-secretors Le^a−b-^)	HPLC−CHIP/TOF MS	Prevalence of fucosylated HMOs in secretors; Lower amounts of fucosylated HMOs in non-secretor individuals	Secretor status influences HMO expression more than the Lewis status
Bode L. et al. [15], 2012	81 BM HIV positive that transmitted the virus vs. 86 BM HIV that did not transmitted the virus vs. 36 BM HIV negative	HPLC	↑ non-3′-SL protection against HIV transmission	Good study population, support the hypothesis of the protective effect of some HMOs against HIV transmission
Smilowitz et al. [126], 2013	52 healthy women (34–38 GW)	^1^H-NMR	↑ 2′-FL, LDFT and Lacto-N-fucopentose in BM of mothers with secretor phenotype	Differentiation between the two phenotypes according to the type of HMOs detected
Praticò et al. [123], 2014	20 healthy women	^1^H-NMR	Se+/Le+: fucosylated HMOs Se-/Le+: absence of alpha-1,2-fucosylated HMOs Se-/Le-: absence of LNFP-II, LNDFH-I and LNDFH-II	Metabolomics approach linked the phenotype (3 different classes) with HMO content of the milk Different HMOs could impact the infant gut microbiota differently
Van Leeuwen et al. [127], 2014	32 breast-feeding mothers	^1^H-NMR	Absence of alpha-1-2-fucosylated HMOs in Non-secretors Abundance of α1-2 fucosylated HMOs (2′-FL) in Secretors	Different Se/Le genotypes correlate to different patterns of HMOs (Group 1–4)
Alderete et al. [128], 2015	31 mother–infant pairs	HPLC	LNFP-II and DSLNT correlate with greater fat mass LNFP-I was inversely associated with infant weight and fat mass	Explores the correlation among HMO composition and infant growth and obesity during the first 6 months of life
Spevacek et al. [129], 2015	15 BM preterm vs. 13 BM preterm	^1^H-NMR	↑ 3-FL during the 28 days of lactation in both milk ↑ LNFP and LNnT in preterm C at the beginning of lactation	HMO production is finely regulated by the mammary gland Dynamic composition in both milk in the first month of lactation Preterm milk varies the most
Sundekilde et al. [130], 2016	15 BM preterm vs. 30 at term	^1^H-NMR	↑ Lacto*-N*-tetraose, LNDFH-I, 3′-SL, and 6′-SL in preterm	First study comparing preterm vs. at term milk in a lactation period (up to 14 weeks) Metabolomics linked to clinics These HMOs may be the cause of low incidence of NEC in breastfed preterms
Charbonneau et al. [131], 2016	88 Malawian breast-feeding mothers (69 secretors, 19 non-secretors) and their newborns (59 with severe stunting, 29 healthy) 215 Malawian breast-feeding mothers (155 secretors, 60 non-secretors)	LC-TOF MS	Among secretors, no significant differences in HMO profiles; Among non-secretors, fucosylated and sialylated HMOs were more abundant in milk from mothers who delivered healthy infants compared to those who delivered stunted children	Lower fucosylated and sialylated HMOs in milk of mothers with severely stunted infants Breast milk from non-secretor Malawian mothers might be less supportive for their children’s growth
McGuire et al. [132], 2017	410 BM healthy women from different geographical location	HPLC	↑ 3-FL in Swedish mothers ↑ DSLNT in Gambian mothers	HMOs vary in different locations around the world
Dessì et al. [133], 2018	53 BM of healthy mothers	^1^H-NMR	2′-FL. LDFT. LNFP-I secretor phenotype 3-FL and LNFD III non -secretor phenotype	HMO content differentiated in secretor and non-secretor phenotype
Gomez-Gallego et al. [134], 2018	79 BM of healthy mothers from Finland, Spain, South Africa, China (vaginal birth vs. c-section)	^1^H-NMR	↑ LNFP-I and 2′-FL in Finland and Spain ↑ 3-FL and LNFP-III in South Africa and China	The effect of c-section on BM is different depending on the location Different HMO production among the geographical location may results in different roles for human milk in relation to infant health and development.
Samuel et al. [40], 2019	331 European mothers from Spain, France, Italy, Norway, Portugal, Romania and Sweden 290 of 331 also tested for ppBMI correlation (229 normal, 61 overweight)	*LC*-*FLD* H-NMR	Highest concentration of 2′-FL, LNFP-I and DFLNH in Group 3 and 1 Highest concentration of LNFP-II and LNnFP-V in Group 2 Highest concentration of LNFP-III and LNT in Group 4	Different Se/Le genotypes correlate to different patterns of HMOs (Group 1–4) Lower concentrations of LNT, LNnT and LNFP-V and higher concentration of 3′-SL, 6′-GL in overweight women
Wang et al. [39], 2021	10 breast-feeding mothers	^1^H-NMR	2′-FL and LNFP-I in milk from Secretors, absent in non-secretors. LNFP-II, 3-FL, LDFT, and LNFP-III in milk of Lewis positive, absent in Lewis negative. Significant differences exist in the non-HMO milk metabolome of non-secretor, Lewis negative mothers compared to other phenotypes.	Different Se/Le genotypes correlate to different patterns of HMOs (Group 1–4) Mammary gland metabolome is globally influenced by the inactivity of FUT2 and FUT3: Se–Le– phenotype shows a different non-HMO metabolome compared to other phenotypes
Saben JL et al. [135], 2021	194 mother–child pairs (Mothers classified by BMI: 68 NW, 51 OW, 75 OB) 155 infants tested for daily HM intake	HPLC	Among non-secretors, significantly lower concentration of 3′-SL in OW compared to NW. Among secretors, lower concentration of LNT, LNnT in OW compared to NW; higher LNnT and 3-FL in OB women compared to NW and OW.	Composition in terms of HMOs might be influenced by maternal weight, especially in non-secretor women. Infants born to OB women consumed fewer LNH, DFLNH and other HMOs compare to those born to OW and NW mothers, while they presented the highest consumption of LNnT

BM: breast milk, HM: human milk, FM: formula milk, C: colostrum, TM: transition milk, TM: at term milk, WG: week of gestation, CD: Cesarean delivery, SD: spontaneous delivery, LC-MS: liquid chromatography, GC-MS gas chromatography, CE-MS: capillary electrophoresis ^1^H NMR nuclear magnetic resonance spectroscopy, MRS Proton magnetic resonance spectroscopy, HPLC High-performance liquid chromatography, LC CHIP/TOF MS Liquid Chromatography chip/Time-Of-Flight Mass Spectrometry, LC-FLD Liquid Chromatography with fluorescence detection, HPAEC High-performance anion-exchange chromatography, LNFP-II Lacto-*N*-fucopentaose II, LNDFH-II Lacto-*N*-difucohesaose II, ↑: higher concentration of the HMOs, OW overweight, NW normal weight, OB obese. ↓: lower concentration of the HMOs, OW overweight, NW normal weight, OB obese.

## Data Availability

Not applicable.

## List of Abbreviations

HMOs	Human Milk Oligosaccharide
SCFAs	Short-Chain Fatty Acids
Gal	β-d-galactose
Glc	β-d-glucose
GlcNAc	β-d-*N*-acetyglucosamine
Fuc	α-l-fucose
Sia	sialic acid α-d-*N*-acetylneuraminic acid
LNnT	Lacto-*N*-neotetraose
LNT	Lacto-*N*-tetraose
LNT-II	Lacto-*N*-triose II
2′-FL	2′-fucosyllactose
3-FL	3-fucosyllactose
3′-SL	3′-sialyllactose
6′-SL	6-’sialyllactose
LNFP-I	Lacto-*N*-fucopentaose I
LNFP-II	Lacto-*N*-fucopentaose II
LNFP-III	Lacto-*N*-fucopentaose III
Th2	T helper 2 lymphocyte
IL-4	Interleukin-4
IFN-γ	Interferon gamma
IgE	Immunoglobulin E
Th	T helper
DSLNT	disialyllacto-*N*-tetraose
LDFT	Lactodifucotetraose
LNDFH-I	Lacto-*N*-difucoesaose I
LNDFH-II	Lacto-*N*-difucohesaose II
DFLNH	difucosyllacto-*N*-hexaose
LNH	Lacto-*N-*hexaose
LNnFP-V	Lacto-*N*-neofucopentaose V
LNF-I	Lacto-*N-*fucopentaose
3′-GL	3′-galactosyllactose
6′-GL	6′-galactosyllactose
MAPK	Mitogen-Activated Protein Kinase
TRL	Toll Like Receptor
Sieglecs	sialic acid binding Immunoglobulin-like lectins
APC	Antigen Presenting Cells
TNF-α	Tumor Necrosis Factor alpha
NEC	necrotizing enterocolitis
HIV	human immunodeficiency virus
LC	lectin receptors
